# Trends in psychosomatic symptoms among adolescents and the role of lifestyle factors

**DOI:** 10.1186/s12889-024-18327-x

**Published:** 2024-03-21

**Authors:** Benti Geleta Buli, Susanna Lehtinen-Jacks, Peter Larm, Kent W. Nilsson, Charlotta Hellström-Olsson, Fabrizia Giannotta

**Affiliations:** 1 Department of Public Health Sciences, Mälardalen University, 721 23 Västerås, Box 883, Sweden; 2 Department of Public Health Sciences, Stockholm University, Stockholm, Sweden; 3 Center for Clinical Research, Uppsala University, Västmanland County Hospital, Uppsala, Sweden; 4 Department of Neuroscience, Uppsala University, Uppsala, Sweden; 5 Department of Psychology, University of Turin, Turin, Italy

**Keywords:** Adolescent, Mental health, Psychosomatic symptoms, Lifestyle factors, Trends, HBSC

## Abstract

**Background:**

Adolescent mental health problems are on the rise globally, including in Sweden. One indicator of this trend is increased psychosomatic symptoms (PSS) over time. Lifestyle factors such as physical activity (PA), diet, smoking, and alcohol consumption may influence the time trends in PSS; however, the evidence base is scarce. The aim of this study was to investigate associations between time trends in PSS and lifestyle factors.

**Methods:**

The study was based on data collected from a nationally representative sample of 9,196 fifteen-year-old boys and girls in Sweden using the Health Behavior in School-aged Children (HBSC) symptom checklist. The sample comprised nearly equal proportions of girls (50.5%) and boys. The lifestyle factors examined in this study included PA, regular breakfast intake, consumption of fruits, vegetables, sweets, or soft drinks, smoking, and alcohol drunkenness. We used data from 2002 to 2018 and stratified by family affluence scale (FAS) to demonstrate how the associations varied among the FAS groups. We fitted separate regression models for the high- and low-FAS groups, where interaction terms between the year of survey and each lifestyle factor were used to estimate the level and direction of associations between the factors and trends in PSS.

**Results:**

There was a generally increasing trend in PSS mean scores from 2.26 in 2002 to 2.49 in 2018 (*p* <.001). The changes in each survey year compared to the average mean scores during the preceding years were significant in all years except 2010. Regular breakfast intake, daily fruit and vegetable consumption, and higher PA were associated with lower PSS mean scores, while smoking and drunkenness had opposite associations with PSS. The only significant interaction between survey year and the lifestyle factors was observed regarding drunkenness in the high FAS group, suggesting that the association between trends in PSS and the experience of getting drunk at least twice got stronger over time (B = 0.057; CI:0.016, 0.097; *p* <.01).

**Conclusions:**

The results indicate increasing trends in PSS among young people in Sweden from 2002 to 2018, with a significant increase observed among adolescents in the high FAS group who reported getting drunk on at least two occasions.

**Supplementary Information:**

The online version contains supplementary material available at 10.1186/s12889-024-18327-x.

## Introduction

Adolescents are burdened with a heavy load of mental health problems [1], despite being perceived as healthy [2]. Increasing trends of these problems have been reported both in Sweden and elsewhere [3–5] in recent decades. One indicator of these trends is an increase in psychosomatic symptoms (PSS) over time [3, 6, 7]. The causes of these increased trends remain largely unknown. This study aims to illuminate factors associated with these trends by grounding itself in the social determinants of health model (SDH) [8, 9]. The SDH, rooted in social epidemiology theory [10], posits that health or disease results from a complex interplay of conditions broadly categorized into (a) structural determinants, such as social and political context, gender, income, ethnicity, and (b) intermediate determinants, including individual lifestyle behaviors. While previous studies have explained the trends in mental health problems from various perspectives, such as migration background [11], gender and school-related stress [12], and socioeconomic status (SES) [13], none have explored the potential role of lifestyle behaviors in influencing these trends. This study aims to fill that gap.

Previous studies have demonstrated that lifestyle behaviors can have positive or negative impacts on mental health. For example, frequent and regular consumption of fruits and vegetables, engaging in regular PA, and regular breakfast intake have been linked to improved mental health outcomes [14–18]. Conversely, frequent consumption of sweets and sugar-added drinks, physical inactivity, alcohol drunkenness, and substance abuse, such as tobacco, are associated with PSS and other poor mental health conditions [15, 19–23]. The mechanisms that may link these behaviors to mental health are varied. For instance, one hypothesized pathway between unhealthy dietary habits and mental health issues is through the development of obesity, which can trigger adipose-induced inflammation and lead to mental health problems such as depression [24]. Alcohol exposure, for example, episodic heavy drinking [25], during adolescence disrupts brain development and executive function maturation, which negatively impacts emotional states [26]. Moreover, smoking is associated with changes in brain structure and neural circuitry in brain regions, and these changes are implicated in mental disorders [27]. Studies have also shown that PA improves self-esteem, cognitive performance, and educational achievements, which, in turn, have positive associations with mental health [14]. Although the associations between lifestyle factors and mental health problems including PSS are well documented, little is known about the influence of these factors on trends in PSS among adolescents. On the other hand, studies have demonstrated reciprocal relationships between mental well-being and lifestyle behaviors. Mental health problems have been associated with unhealthy eating [28, 29], an increased risk of smoking [30], alcohol use disorder [31], and a sedentary lifestyle. However, investigation of the directions the association is beyond the scope of this study.

Furthermore, studies also show that lifestyle behaviors vary across socioeconomic gradients. Adolescents from lower SES groups show poorer diets, less PA, and increased cigarette smoking than their peers from higher SES groups [32, 33]. Thus, it is important to take SES into consideration when examining relations between lifestyle factors and PSS. In this study, we use Family Affluent Scale (FAS) as an indicator of the family’s economic status.

The present study therefore aims to investigate the association between trends in PSS from 2002 to 2018 and lifestyle factors, including PA, breakfast regularity, consumption of fruits, vegetables, sweets or soft drinks, smoking, and alcohol drunkenness over time among adolescents in thigh- and low-FAS groups. This study utilizes the exposure and vulnerability hypotheses to investigate these associations. The exposure hypothesis guides the examination of whether trends in PSS could be explained by changes in lifestyle factors over time (see [12] and [34]); whereas the vulnerability hypothesis examines whether the strength of associations between trends in PSS and lifestyle factors varies across FAS groups over time [12, 34].

## Methods

### Participants

This study uses data from the Swedish Health Behavior in School-aged Children (HBSC) studies conducted in 2002, 2006, 2010, 2014 and 2018. These studies collect data from nationally representative samples of 11-, 13-, and 15-year-old boys and girls through cross-sectional surveys to track the health and lifestyle of adolescents with a focus on their social and environmental contexts [35]. The HBSC data were collected using a two-stage cluster design with schools being primary sampling units. Once a school was selected, one class per grade/age group (grade 5 (11-year-olds), grade 7 (13-year-olds) or 9 grade (15-year-olds)) was selected from each cluster where data were collected from all students in that class under the supervision of the teacher (see Högberg et al. [12] for a detailed description of the process). This study focuses on data from the 15-year-olds, with nearly equal proportions of girls (50.5%) and boys. Table 1 presents the number of participants by sex and SES. The participation of schools was reasonably high across the survey years, except in 2018 when it was only 47%. Students’ response rates were high in all years, ranging from 81 to 88% [36].

**Table 1:** Description of the 15-year-old participants in the Swedish HBSC 2002–2018, by sex and FAS^†^.

	2002 n (%)	2006 n (%)	2010 n (%)	2014 n (%)	2018 n (%)
Boys	609 (50)	752 (49)	1059 (51)	1358 (49)	771 (48)
Girls	609 (50)	774 (51)	1031 (49)	1408 (51)	825 (52)
Low FAS	299 (25)	270 (18)	280 (14)	609 (23)	311 (20)
High FAS	918 (75)	1250 (82)	1790 (86)	2102 (77)	1280 (80)
Schools response (%) *	84	83	88	77	47
Students response (%) *	84	81	86	88	87

^†^FAS = Family Affluence Scale is a composite of four measures of family affluence (family car ownership, own bedroom, family holidays, and family computer ownership) [37], and dichotomized in each wave of data using standard deviations (low FAS; ≤ -1 SD, high FAS; > -1 SD)

* Public Health Agency of Sweden. Skolbarns Hälsovanor 2017/18-Grundrapport [In English: Health Behavior in School-Aged Children 2017/18; Basic Report]. Östersund: Public Health Agency of Sweden; 2018. Report No.: 18,065

### Psychosomatic symptoms

We measured psychosomatic symptoms (PSS) using a scale developed from the HBSC symptoms checklist, designed as a nonclinical measure of health complaints. Participants were asked if and how often they had experienced the following eight symptoms in the past six months: headache, stomachache, backache, feeling low, feeling irritable, feeling nervous, sleeping difficulties, and feeling dizzy. The response to each question ranged from “daily” to “rarely/never” and was coded as “about every day” =1, “more than once a week” = 2, “about every week” = 3, “about every month” = 4, and “rarely/never” = 5. This measure has shown acceptable reliability and internal consistency among adolescents of the same age in Canada [38]. We reverse coded the items and created a composite variable PSS from the mean of the scores, where higher mean scores signified poorer mental health of a participant.

### Breakfast regularity

This variable is constructed from school children’s response to the following question: “On weekdays, how often do you usually have breakfast (more than a glass of milk or fruit juice)?”. The response categories include “I never have breakfast during weekdays”, “1 day”, “2 days”, “3 days”, “4 days”, and “5 days”. While specific guidelines on regularity of breakfast do not exist, we adopted an approach by Pedersen et al. [39] to dichotomize the variable into “regular” for those who reported having breakfast at least 3 days in a week and “irregular” for those who reported having breakfast less than 3 days a week.

### Dietary habits

Habits of consuming different food items were asked using the following question: “How many times a week do you usually eat or drink, a) fruits, b) vegetables, c) sweets, or d) soft drinks?”. The response categories include “never”, “less than once a week”, “once a week”, “2–4 days a week”, “5–6 days a week”, “once daily”, and “more than once daily”. We first reverse coded sweets and soft drinks so that low intake favors positive health according to previous studies [18]. We then dichotomized the intake habit of each of the four food items into 0 or 1 using cutoff points reported in Kleppang et al. [18], a study that used a similar dataset. Daily or more than once daily intake of fruits and vegetables, as well as less or equal to four days a week intake of sweets or sugar-added soft drinks, were each coded as (1). Less than daily intake of fruits and vegetables and intake of sweets or sugar-added soft drinks for more than four days in a week were each coded (0). A composite variable was created from these four food items, giving a scale of 0–4. We finally dichotomized this scale at a median value where values 0–2 were categorized as “unhealthy” (0) and 3–4 as “healthy” (1) following the experience of Kleppang et al. [18]

### Physical activity (PA)

PA was assessed based on the question: “Over the past 7 days, on how many days were you physically active for at least an average of 60 minutes per day?” We used the WHO-recommended level of PA for children, which states that children should accumulate an average of 60 min of moderate to vigorous physical activities (MVPA) daily [40]. Following the experience of a previous study [41], we categorized participants who were active for at least five days as “sufficiently active” and those who were active for less than five days as “insufficiently active.”

### Smoking

Participants were asked the question: “How often do you smoke tobacco at present?”, with response categories including “I do not smoke”, “Less than once a week”, “At least once a week but not every day”, or “Daily”. This question was asked differently in 2018, and we used the question that asked: “On how many days (if any) have you smoked cigarettes in the last 30 days?” with response categories of “Never”, “Once”, “2–3 days”, “4–10 days” or “More than 10 days”. We created a binary variable by combining these two questions where those never smoked/do not smoke were coded (0) and the rest were coded (1).

### Alcohol drunkenness

We used alcohol drunkenness in this study instead of mere frequency of alcohol consumption, as the former was found to have an association with mental health [42]. The following question was presented to the participants: “Have you ever had so much alcohol that you were really drunk?”, with response categories including “Never”, “Once”, “2–3 times”, “4–10 times” or “More than 10 times”. Following recommendations from previous studies [43, 44], we coded individuals who never got drunk or got drunk only once as (0), and the rest as (1).

### FAS

This study used the 2001/2002 version of the FAS to measure family’s economic status. The scale was developed for HBSC surveys as an alternative measure of family wealth and has been used for over two decades [37, 45, 46]. The scale comprises four items that include family car ownership, bedroom occupancy, family holidays, and computer ownership.

- *Does your family own a car, van, or truck?* Response categories were No (0), Yes, one (1), Yes, two or more (2).
- *Do you have your own bedroom for yourself?* Response categories were No (0) and Yes (1).
- *During the past 12 months, how many times did you travel away on holiday with your family?* Response categories were as follows: not at all (0), once (1), twice (2), and more than twice (3).
- *How many computers does your family own?* Response categories were: None (0), One (1), Two (2), More than two (3).

A composite FAS was calculated for each adolescent by summing the scores of each item, and the resulting scale ranged from 0 to 9. The scale was then dichotomized as low or high FAS in each wave of data using standard deviations. Considering the country’s overall economic changes [47], rather than employing a dichotomy with pooled data from all waves, we conducted the dichotomy for each wave of data. This approach aimed to minimize the risk of misclassification as low or high FAS. In this categorization, values equal to or less than − 1 SD were classified as low FAS (0), while values greater than − 1 SD were considered high FAS (1).

### Statistical analysis

Two types of analyses were conducted. The first was identifying time trends both in PSS and lifestyle factors by comparing pooled mean scores for PSS and proportions for the categorical lifestyle variables at each survey year. Reverse Helmert contrast [48] was used to compare the mean score or proportion in a given survey year with the average of mean scores or proportions of the preceding survey years. Hence, we compared 2006 vs. 2002, 2010 vs. average of 2002 and 2006, 2014 vs. average of 2002, 2006 and 2010, and finally 2018 vs. average of all preceding survey years. Cohen’s d was used for measuring the effect sizes [49] of the differences in the mean PSS scores over time and the years during which significant changes were observed are marked with asterisks (*) in relevant tables.

The second type of analysis was fitting multiple linear regression models to investigate the associations between trends in the dependent variable PSS and the independent variables breakfast regularity, dietary habits (unhealthy/healthy), PA, smoking, and alcohol drunkenness. In Model 1, in addition to assessing the main effects of each independent variable on the trends in PSS, we also tested exposure hypothesis by examining the mediation effects of each lifestyle factor on the relationship between survey year and the outcome, using the approach outlined in Karlson et al. [50]. In Model 2, the study assessed the effects of the interaction between time (survey year) and each independent variable with the aim to test vulnerability. The coefficients of the interaction terms were used to estimate the level and direction of associations between the trend in PSS and the independent variables. R-squared change was used to assess the significance of the difference between Models 1 and 2 in predicting trends in PSS following the inclusion of the interaction terms. The data were stratified by FAS to show the trends in the low and high FAS groups separately. The results were adjusted for sex.

## Results

### Time trends in psychosomatic symptoms from 2002 to 2018

Table 2 shows that PSS mean scores exhibited increasing trends from 2002 (2.26) to 2006 (2.33), from 2010 (2.25) to 2014 (2.40), and finally from 2014 (2.40) to 2018 (2.49). The Helmert reverse contrast confirms that the mean PSS scores for a given survey year were significantly higher than the means of all preceding years, except for 2010, when it was only marginally significant. The overall changes were significant (F = 23.858, df = 4, *p* <.001), although the effect sizes were very small (Cohen’s d ranging from 0.09 to 0.17).

**Table 2:** Mean PSS scores by year of survey, sex, and FAS, compared using reverse helmert contrasts‡

Year of survey	Psychosomatic symptoms, mean scores, and SE (in bracket)				
	Total	Boys	Girls	Low FAS	High FAS
2002	2.26 (0.024)	2.02 (0.031)	2.50 (0.033)	2.39 (0.052)	2.22 (0.027)
2006	2.33 (0.021)*	2.08 (0.028)	2.58 (0.030)	2.46 (0.055)	2.31 (0.023)*
2010	2.25 (0.018)	2.00 (0.023)	2.50 (0.026)	2.38 (0.054)	2.23 (0.019)
2014	2.40 (0.016)***	2.10 (0.021)*	2.68 (0.022)***	2.42 (0.037)	2.39 (0.018)***
2018	2.49 (0.021)***	2.20 (0.028)***	2.76 (0.029)***	2.52 (0.052)	2.48 (0.022)***

***/**/* = *p* <.001/0.01/0.05, respectively. PSS = Psychosomatic symptoms; SE = Standard error Family Affluence Scale is a composite of four measures of family affluence (family car ownership, own bedroom, family holidays, and family computer ownership) [37], dichotomized in each wave of data using standard deviations (low SES; ≤ -1 SD, high SES; > -1 SD)

**Total**: F = 23.858, df = 4, *P* <.001, eta^2^ = 0.01; **Boys**: F = 8.157, df = 4, *P* <.001, eta^2^ = 0.007; **Girls**: F = 16.783, df = 4, *p* <.001, eta^2^ = 0.014; **Low FAS**: F = 1.241, df = 4, *p* =.291, eta^2^ = 0.003; **High FAS**: F = 24.911, df = 4, *p* <.001, eta^2^ = 0.013

‡Reverse Helmert Contrast is a statistical technique that compares a value of a variable at given level with the mean of the previous level(s) [48]

Regarding sex differences, girls had significantly higher mean PSS scores than boys across all years (Cohen’s d ranging from 0.62 to 0.71; *p* <.001). For changes over time, the trends in PSS scores began to increase significantly for both girls (F = 16.783, df = 4, *p* <.001) and boys (F = 8.157, df = 4, *p* <.001) after 2010 (Table 2). A significant interaction term between sex and the year of survey (B = 0.029; *p* =.026) indicates that the increase was more pronounced among girls than boys (Model 2 in Supplementary Table 1). When comparing adolescents across FAS gradients, those with high FAS had significantly lower mean PSS scores than those with low FAS (Model 1 in Supplementary Table 1), especially during the period 2002–2014 (Table 2), with generally small effect sizes (Cohen’s *d* ranging from 0.03 to 0.22, *p* <.01). There was no significant difference in the mean PSS scores between the FAS groups in 2018. For changes over time, a significant interaction term between FAS and year of survey (B = 0.036; *p* =.021) indicates that an increase in FAS is associated with significantly increasing trends in the mean PSS scores over time (Model 2 in Supplementary Table 1). The results in Table 2 further elaborate that the trend in PSS significantly increased in 2006 and after 2010 in the high FAS group (F = 24.911, df = 4, *p* <.001), while there was no significant change in the low FAS group (F = 1.241, df = 4, *p* =.291).

### Time trends in lifestyle factors from 2002 to 2018

In general, the analysis of trends in lifestyle factors yielded mixed results. As presented in Table 3, positive behaviors (fruit and vegetable consumption, and physical activity) either remained stable or slightly increased over the survey period, except for breakfast intake. The latter showed an overall decrease during the study period, with a significant change occurring in 2018, when it decreased by 3% (*p* <.01). Stratification by FAS showed that the decrease in regular breakfast intake occurred in the low FAS group during the same period. However, consumption of vegetables showed a consistently significant increase across the survey years, except in 2010 when the increase was not significant. On the other hand, negative behaviors (consumption of sweets and soft drinks, smoking, and alcohol drunkenness) significantly decreased over time (*p* <.05), except for sweets and soft drinks, which remained stable or increased, respectively, for those in the low FAS group. The prevalence of alcohol drunkenness exhibited a substantial overall decrease by -70.5% (*p* <.001) between 2002 and 2018. This decline was statistically significant in both the low FAS group (-65.8%, *p* <.001) and the high FAS group (-71.9%, *p* <.001).

**Table 3:** Description of the 15-year-old adolescents’ lifestyle behaviors and their changes over years from 2002 to 2018, in Total and by FAS§

	2002 (%) (X) (Reference)	2006 (%)	2010 (%)	2014 (%)	2018 (%) (Y)	Difference in %^†^
Regular breakfast (Total)	81.8	80.5	79.8	79.4	77.1**	**-5.7**
Low FAS	74.4	74.9	69.6	74.2	63.5***	**-14.7**
High FAS	84.0	81.7	81.5	81.0	80.3	**-4.4**
Physically active (Total)	31.7	34.3	54.9***	38.6	36.8*	**16.1**
Low FAS	27.0	28.2	47.2***	31.8	26.7*	-1.1
High FAS	33.3	35.4	56.2***	40.7	39.2	**17.7**
‡Healthy dietary habit (Total)	35.5	35.0	34.4	36.1	38.7*	**9.0**
Low FAS	37.1	33.1	29.7	36.1	35.9	-3.2
High FAS	35.0	35.4	35.2	36.3	39.3*	**12.3**
Fruits (Total)	22.1	27.9 ***	24.3	21.4**	23.6	6.8
Low FAS	20.4	27.1	20.6	19.0	23.8	16.7
High FAS	22.7	28.2**	24.9	22.1**	23.7	4.4
Vegetables (Total)	29.7	34.2 *	34.4	38.6***	42.0**	**41.4**
Low FAS	25.4	33.1*	30.1	32.8	34.4	**35.4**
High FAS	31.2	34.4	35.1	40.4***	43.8***	**40.4**
Sweets (Total)	19.2	19.2	20.5	18.8	14.3***	**-25.5**
Low FAS	19.8	16.4	24.5*	22.4	21.0	6.1
High FAS	19.0	19.8	19.8	17.5	12.7***	**-33.2**
Soft drinks (Total)	15.8	19.8**	20.1*	18.8	14.1***	**-10.8**
Low FAS	14.4	17.1	20.6	18.9	18.1	**25.7**
High FAS	16.2	20.4	20.0	18.7	13.0	**-19.8**
Smoking (Total)	23.8	13.0***	20.2	11.3***	11.5***	**-51.7**
Low FAS	21.8	11.3***	24.7**	11.7***	13.1	**-39.9**
High FAS	24.4	13.3***	19.4	10.8***	11.1***	**-54.5**
Drunkenness (Total)	38.6	26.1 ***	24.0***	16.4 ***	11.4***	**-70.5**
Low FAS	34.8	22.2***	20.1**	14.9***	11.9***	**-65.8**
High FAS	39.9	26.9***	24.7***	16.8***	11.2***	**-71.9**

***/**/* = indicate significant proportion difference between the value at a specific survey year & means of the previous years at *p* <.001/0.01/0.05, respectively. Family Affluence Scale is a composite of four measures of family affluence (family car ownership, own bedroom, family holidays, and family computer ownership) [37], and dichotomized in each wave of data using standard deviations (low SES; ≤ -1 SD, high SES; > -1 SD)

§ Reverse Helmert Contrast was used to estimate the changes in the proportion of adolescents’ lifestyle behaviors over the years of survey. Reverse Helmert Contrast is a statistical technique that compares the value of a variable at given level with the mean of the previous level(s) [48]

†The difference is calculated using the formula: $\left(\frac{(Y-X)}{X}*100\right)$ and significant differences are presented in **bold**

‡Healthy dietary habit is a composite indicator from: fruits, vegetables, sweets & soft drinks

### Association between trends in PSS and lifestyle factors

Table 4 presents regression results on the associations between trends in PSS and lifestyle factors in the low and high FAS groups. Model 1 shows associations without interaction effects, and Model 2 shows the effects of interaction between lifestyle factors and years of survey.

### Cross-sectional associations

In Table 4, Model 1, within both FAS groups, regular breakfast consumption was linked to lower PSS mean scores, while smoking and alcohol drunkenness were associated with higher PSS mean scores. PA was statistically significantly associated with lower PSS mean scores only in the high FAS group.

### Exposure hypothesis

Mediation analysis revealed that incorporating lifestyle factors into the regression model led to the identification of mediating effects of these factors, as evidenced by the difference between the direct and total effects of the survey year. The mediation analysis among the total study sample (both low and high FAS groups) shows significant indirect effects through the lifestyle factors (B = − 0.0184, *p* <.001) (Supplementary Fig. 1a). Further analysis by stratifying the sample by FAS demonstrated that the significance of the mediating effects of the lifestyle factors holds true for adolescents in both the low FAS (B = − 0.0185, *p* <.01) (Supplementary Fig. 1b) and high FAS (B = − 0.0184, *p* <.001) groups (Supplementary Fig. 1c). However, the proportion mediated by each lifestyle factor in each FAS group varies slightly, as indicated in Supplementary Fig. 1b and 1c.

### Vulnerability hypothesis

**Fig. 1:**
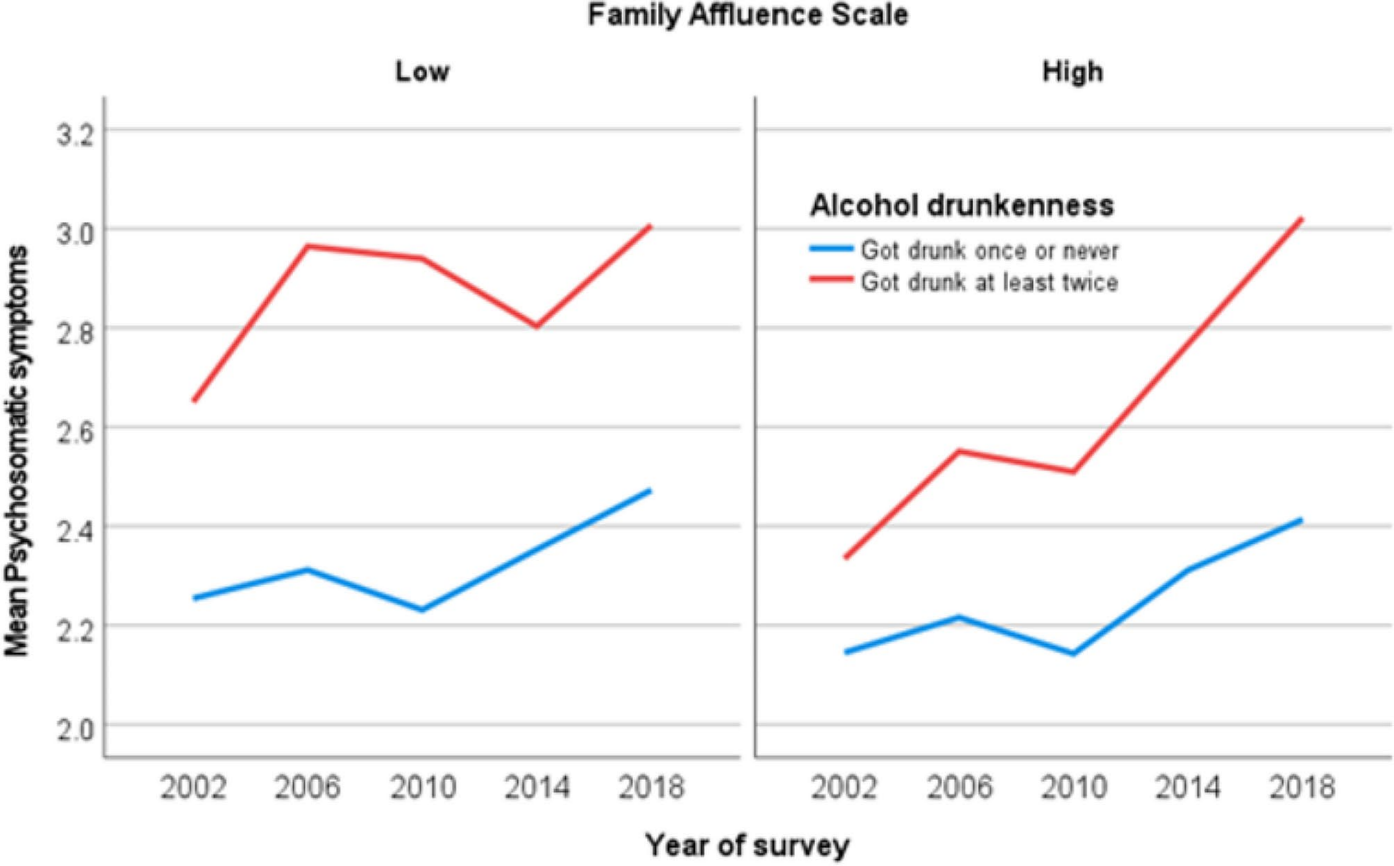
Trends in psychosomatic symptoms among 15-years old adolescents by family affluence scale and alcohol drunkenness

The results in in Model 2 of Table 4 reveal that the interaction between alcohol drunkenness and year of survey in the high FAS group was the only significant factor (B = 0.057; CI:0.016,0.097; *p* <.01), suggesting that alcohol drunkenness was the only lifestyle factor in this study that is statistically significantly associated with the trends in PSS over time. The results indicated that the positive association between getting drunk with alcohol at least twice and trends in PSS has become stronger over time among adolescents in the high FAS group (see also Fig. 1).

**Table 4:** Multiple linear regression results of the association between lifestyle factors and mean PSS scores among 15-year-old adolescents from 2002–2018 in low and high FAS groups

		Low FAS (a) (*N* = 1,769)			High FAS (b) (*N* = 7,340)	
	B	Lower Bound	Upper Bound	B	Lower Bound	Upper Bound
**Model 1**						
Survey year	0.040**	0.010	0.071	0.080***	0.066	0.094
Girl	0.468***	0.386	0.549	0.502***	0.466	0.538
Regular breakfast	− 0.258***	− 0.350	− 0.165	− 0.233***	− 0.281	− 0.186
Dietary habit	0.059	− 0.026	0.143	0.041*	0.004	0.078
Physical activity	− 0.046	− 0.133	0.041	− 0.091***	− 0.128	− 0.055
Smoking	0.392***	0.261	0.523	0.280***	0.221	0.340
Alcohol drunkenness	0.274***	0.153	0.394	0.177***	0.125	0.228
**Model 2**						
Survey year	0.044	− 0.025	0.113	0.090***	0.051	0.129
Girl	0.468***	0.386	0.550	0.501***	0.465	0.536
Regular breakfast	− 0.232	− 0.474	0.011	− 0.147*	− 0.279	− 0.015
Dietary habit	0.089	− 0.128	0.306	0.052	− 0.049	0.154
Physical activity	− 0.076	− 0.310	0.157	− 0.084	− 0.186	0.017
Smoking	0.394*	0.072	0.716	0.268***	0.118	0.418
Alcohol drunkenness	0.234	− 0.043	0.512	0.013	− 0.114	0.141
Reg. breakfast * Year	− 0.008	− 0.077	0.060	− 0.026	− 0.063	0.012
Dietary habit * Year	− 0.010	− 0.072	0.053	− 0.003	− 0.032	0.026
Physical activity * Year	0.009	− 0.058	0.076	− 0.003	− 0.032	0.026
Smoking * Year	− 0.001	− 0.098	0.095	0.004	− 0.042	0.050
Alc. drunkenness * Year	0.014	− 0.072	0.100	0.057**	0.016	0.097

***/**/* = *p* <.001/0.01/0.05, respectively; B = unstandardized beta coefficients, Family Affluence Scale is a composite of four measures of family affluence (family car ownership, own bedroom, family holidays, and family computer ownership) [37], and dichotomized in each wave of data using standard deviations (low SES; ≤ -1 SD, high SES; > -1 SD). PSS = Psychosomatic symptoms

Model Fitness: *Low FAS*: Model 1: R^2^ = 0.175, R^2^-change = 0.176; F(7, 1583) = 48.234, *p* <.001; Model 2: R^2^ = 0.175; R^2^-change = 0.001; F(12,1578) = 28.077, *p* <.001; ***High FAS***: Model 1: R^2^ = 0.179, R^2^-change = 0.177; F(7, 6759) = 209.006; *P* <.001; Model 2: R^2^ = 0.178; R^2^-change = 0.002; F(12, 6754) = 123.209; *p* <.001

## Discussion

The primary objective of this study was to investigate the influence of lifestyle factors on the trend in PSS among Swedish adolescents from 2002 to 2018. Additionally, the study aimed to explore how these relationships between PSS trends and lifestyle factors differed across varying levels of FAS. First and foremost, this analysis revealed a significant increase in time trends in PSS, particularly after 2010, which aligns with prior research in Sweden [3, 6], as well as several other countries across Europe and North America, Israel, and New Zealand [7]. However, the trends differed for adolescents in the high and low FAS groups. Despite consistently higher PSS scores among the adolescents in the low FAS group, the increase over time was significant only among those in the high FAS group. This finding contrasts with prior research that reported more significant increasing trends in mental health problems, including PSS, among adolescents in the low SES group compared to those in the high SES group [51, 52]. This difference could arise, among other things, from the fact that this study employed objectively measured economic indicator, FAS, whereas the referenced studies used subjective SES measures linked to individuals’ self-perceived social standing.

This study revealed generally increasing or stable positive behaviors and decreasing or stable negative behaviors regarding trends in lifestyle factors. Some of the findings are consistent with previous research, while others are not. For instance, the consumption of fruits and regular intake of breakfast remained stable over time, which is consistent with previous studies in similar settings [53, 54]. The study also reveals that there have been significant increases in PA trends, which contradicts previous studies that reported decreasing trends in PA [55] among Swedish adolescents. The discrepancy may stem from the difference in measurement tools used by the two studies. Our study relies on self-reported levels of MVPA, while Conger et al. [55] utilized wearable devices to directly measure activity levels, thus mitigating potential recall and other self-report biases. On the positive side, the significant decrease in negative behaviors such as smoking and drunkenness is consistent with findings from previous studies [56–58]. While the rates of change in fruit and vegetable consumption, smoking, and alcohol consumption were quite comparable between the high- and low-FAS groups, noticeable differences were observed in terms of regular breakfast intake, consumption of sweets or soft drinks, and PA. In the high-FAS group, the proportions of adolescents consuming sweets or soft drinks significantly decreased by 33% and 20%, respectively. In contrast, there was a significant increase in the consumption of sweets (26%) and stability in the consumption of soft drinks among those in the low-FAS group. The proportion of adolescents reporting regular breakfast intake decreased by 15% in the low-FAS group, as opposed to a decrease of only by 4% in the high-FAS group. On the other hand, the proportion of adolescents reporting engagement in PA increased by 18% in the high-FAS group, while the change was negligible among those in the low-FAS group. In the examination of associations between lifestyle behaviors and trends in PSS, mediation analysis, aimed at testing the exposure hypothesis, indicated a notable role of lifestyle factors in slowing down the rate of increase in PSS over time among adolescents in both high and low FAS groups as indicated by a significant difference between total and direct effects of year of survey. Regardless of the contribution of the lifestyle factors, however, increasing trends in PSS were observed among those in the high FAS group. This may indicate that the trends in PSS may not be fully explained by these factors.

Nevertheless, the findings of vulnerability hypothesis test reveal that the positive association between trends in PSS and experience of getting drunk at least twice grew stronger over time only among adolescents in the high FAS group. This is intriguing in two ways: first, why did the association between alcohol drunkenness and trends in PSS grew stronger over time only among those in the high FAS group and not among those in the low FAS group? Second, why was drunkenness associated with an increased trend in PSS over time despite a simultaneous significant decrease in drunkenness? The first question could be explained by attributing the difference to the complex relationship between PSS and drunkenness across different FAS gradients. This complexity could be better understood through the lens of threshold-saturation theory [59, 60], which suggests a nonlinear relationship between the two variables. It is possible that the trend might have leveled off beyond a certain point among those in the low FAS group while continuing to increase among those in the high FAS group. In other words, if we view FAS as a spectrum, at the higher end, any escalation in drunkenness could result in a corresponding increase in the mean PSS score over time. Conversely, at the opposite end of the spectrum, a saturation point might have already been reached, and no further increase could be detected. Another explanation could be that we did not have sufficient statistical power in the low FAS group, which has a sample size of only 1,769, compared to the high FAS group, which has a sample size of 7,340.

The second question could be partly elucidated by considering the hardening theory [61]. According to this theory, when there is a general decline in the use of substances such as alcohol within a population, individuals who continue to engage in drinking behaviors are those who are at greater risk of negative consequences of drinking. The theory suggests that the decrease in alcohol consumption, or alcohol drinking problems in this case, predominantly occurs among individuals with lower levels of addiction. In contrast, those who persist in drinking are likely to be the most entrenched drinkers. Recent studies in Sweden [62] and Australia [63] have highlighted high prevalence of heavy and frequent risky drinking behaviors among affluent youth. The studies attributed the behavior to access to protected spaces away from adult supervision, as well as their considerable purchasing power. These findings may point to where instances of risky drinking are concentrated within the young population. As a result, this group of adolescents may be more susceptible to the adverse effects of alcohol drunkenness, including PSS and other mental health problems, as demonstrated in other studies [20, 22, 23]. However, as no single theory can adequately explain the human environment [64], further investigation is necessary to better understand the underlying reasons and mechanisms behind this phenomenon. Additional research can help explore the complex interactions between FAS, alcohol drunkenness, and PSS among adolescents, providing a more comprehensive understanding of these relationships.

It is important to note the significant change in association between trends in PSS and alcohol drunkenness, while no such relationship was observed with other lifestyle behaviors. This underscores the likelihood that factors beyond lifestyle may play a predominant role in explaining the increasing trend in PSS among Swedish adolescents. Previous studies, for example, have linked trends in PSS to school stress [65], parental support related to school matters, school liking, or the conduciveness of the school physical environment [66]. The former study associated heightened school stress with an elevated risk of PSS, while the latter associated increased parental support, increased school liking, and a conducive school physical environment with a reduction in PSS trends.

This study has some limitations that need to be considered in the interpretation of the results. The primary constraint is the low school response rate in 2018 (47%), potentially introducing bias and influencing changes in PSS. Nevertheless, the Public Health Agency of Sweden [36], the institution responsible for the HBSC data in Sweden, clarified that nonresponse was random, unrelated to school location or type, and should therefore not impact the results. The agency’s report further explains that there was no significant difference in the characteristics of the respondents in 2018 compared to those in previous waves of the HBSC surveys in terms of gender, age, family situation, and country of birth. Inconsistencies in measuring alcohol and cigarette smoking variables affected our choices. We used alcohol drunkenness, which reflected lifetime experience, as it was the only consistently measured alcohol-related variable across all waves. Although it does not necessarily represent the current situation, previous research has suggested that harmful drinking on a single occasion can be associated with mental health problems [20]. Similarly, slight changes in smoking questions led us to employ 30-day smoking experience in 2018, contrasting with the measurement of current experience in other waves. This adjustment could result in an overestimation of the proportion of smokers within that wave. However, it is important to note that this irregularity impacts only 17% of the dataset and may not significantly influence the overall results. On the other hand, it is worth considering that smoking rates might not exhibit significant changes in a mere 30-day interval, as supported by findings from previous studies [56]. Thus, we do not anticipate bias from this irregularity. It is also worth mentioning that the lack of complete data on snus and cannabis limited our analysis of substance use to cigarette smoking and alcohol drunkenness. Regarding dietary habits, the study is based on food consumption frequencies, not the amount consumed. Evidence from previous studies, however, shows a strong correlation between food frequency and intake amount [67], and therefore, this may not have detrimental effects on the results. The absence of data regarding migration status or the country of origin of parents or children within the dataset hindered the examination of the effects of this crucial variable. Finally, due to the cross-sectional survey design, we can only identify associations between PSS and lifestyle factors, without drawing causal conclusions. Nevertheless, previous studies have demonstrated the ability of repeated (cross-sectional) surveys to effectively estimate changes over time [3, 6]. The study’s other strength lies in its analysis of nationally representative data from over 9,000 15-year-old boys and girls, with nearly equal gender representation, and covering a period of more than 15 years. Furthermore, this study can be argued to be the first to examine the association of trends in lifestyle factors with trends in PSS across gradients of FAS. In future studies, it may be valuable to explore the reverse relationship between PSS and lifestyle factors, as this aspect was not addressed within the scope of our study.

## Conclusions

The results of this study indicate increasing trends in PSS among Swedish adolescents from the year 2002 to 2018. Apart from the association between drunkenness and the increasing trend in mean PSS scores among adolescents in the high FAS group, lifestyle factors were not sufficient to explain the increasing PSS trends in this segment of the population. This emphasizes the necessity for additional studies aimed at elucidating the determinants behind these trends and offering valuable insights for the development of suitable policy frameworks.

## Electronic supplementary material

Below is the link to the electronic supplementary material.

Supplementary Material 1

Supplementary Material 2

## Data Availability

The datasets used in this study are available from the corresponding author upon request.

## Abbreviations

FAS: Family Affluence Scale
HBSC: Health Behavior in School-aged Children
MVPA: Moderate to vigorous physical activity
PA: Physical activity
PSS: Psychosomatic symptoms
SES: Socioeconomic status
WHO: World Health Organization
