# Correction: Trends in psychosomatic symptoms among adolescents and the role of lifestyle factors

**DOI:** 10.1186/s12889-025-21524-x

**Published:** 2025-03-10

**Authors:** Benti Geleta Buli, Susanna Lehtinen-Jacks, Peter Larm, Kent W. Nilsson, Charlotta Hellström-Olsson, Fabrizia Giannotta

**Affiliations:** 1 Department of Public Health Sciences, Mälardalen University, Box 883, Västerås, 721 23 Sweden; 2 Department of Public Health Sciences, Stockholm University, Stockholm, Sweden; 3 Center for Clinical Research, Västmanland County Hospital, Uppsala University, Uppsala, Sweden; 4 Department of Neuroscience, Uppsala University, Uppsala, Sweden; 5 Department of Psychology, University of Turin, Turin, Italy

**BMC Public Health (2024) 24:878**

10.1186/s12889-024-18327-x

The original publication of this article was missing the following:

- The keyword “HBSC”.
- The acknowledgement section.

The missing acknowledgement is listed below. The original article has been updated.

