# Supplementary material for: Trends in psychosomatic symptoms among adolescents and the role of lifestyle factors
Source: BMC Public Health. 2024 Mar 21;24:878. doi: 10.1186/s12889-024-18327-x (PMC10958834; doi:10.1186/s12889-024-18327-x)
Supplement: Supplementary file 1 — Supplementary Material 1 [file 12889_2024_18327_MOESM1_ESM.docx]

Trends in psychosomatic symptoms among adolescents and the role of lifestyle factors

Benti Geleta Buli *^, a^, Susanna Lehtinen-Jacks ^a^, Peter Larm ^b^, Kent W. Nilsson ^a, c, d^, Charlotta Hellström-Olsson ^a^, Fabrizia Giannotta ^a, b^

^a^Department of Public Health Sciences, Mälardalen University, Sweden

^b^Department of Public Health Sciences, Stockholm University, Sweden

^c^Center for Clinical Research, Uppsala University, Västmanland County Hospital, Sweden

^d^Department of Neuroscience, Uppsala University, Uppsala, Sweden

***Corresponding author**: [benti.geleta.buli@mdu.se](mailto:benti.geleta.buli@mdu.se)

**Supplementary materials: Mediation analysis**

The following mediation analysis results were produced following the approaches outlined in: Karlson, K. B., Holm, A., & Breen, R. (2012). Comparing Regression Coefficients Between Same-sample Nested Models Using Logit and Probit:A New Method. *Sociological Methodology*, *42*(1), 286-313. <https://doi.org/10.1177/0081175012444861>.

Supplementary Fig. 1a. Direct and indirect effects of year of survey on PSS mean scores through lifestyle factors (regular breakfast, healthy/unhealthy diets, Physical activity (PA), smoking and getting drunk): total sample (low and high Family Affluence Scale (FAS)).

**Note:** Reduced = Total effect, Full = Direct effect, and Difference = indirect effect; P_Diff = percent mediated, P_Reduced = percent of total effect contributed by the specific variable.

Supplementary Fig. 1b. Direct and indirect effects of year of survey on PSS mean scores through lifestyle factors (regular breakfast, healthy/unhealthy diets, Physical activity (PA), smoking and getting drunk): Low FAS group.

Supplementary Fig.1c. Direct and indirect effects of year of survey on PSS mean scores through lifestyle factors (regular breakfast, healthy/unhealthy diets, Physical activity (PA), smoking and getting drunk): High FAS group.
