# Supplementary material for: Trends in psychosomatic symptoms among adolescents and the role of lifestyle factors
Source: BMC Public Health. 2024 Mar 21;24:878. doi: 10.1186/s12889-024-18327-x (PMC10958834; doi:10.1186/s12889-024-18327-x)
Supplement: Supplementary file 2 — Supplementary Material 2 [file 12889_2024_18327_MOESM2_ESM.docx]

Trends in psychosomatic symptoms among adolescents and the role of lifestyle factors

Benti Geleta Buli *^, a^, Susanna Lehtinen-Jacks ^a^, Peter Larm ^b^, Kent W. Nilsson ^a, c, d^, Charlotta Hellström-Olsson ^a^, Fabrizia Giannotta ^a, b^

^a^Department of Public Health Sciences, Mälardalen University, Sweden

^b^Department of Public Health Sciences, Stockholm University, Sweden

^c^Center for Clinical Research, Uppsala University, Västmanland County Hospital, Sweden

^d^Department of Neuroscience, Uppsala University, Uppsala, Sweden

***Corresponding author**: [benti.geleta.buli@mdu.se](mailto:benti.geleta.buli@mdu.se)

Supplementary Table 1. Linear regression results of the associations between year of survey, sex (girls compared to boys), and Family Affluence Scale (FAS) (high compared to low) with psychosomatic symptoms among 15-year-old adolescents in 2002 - 2018.

|  | **B (S.E)** | **p-value** | **Lower Bound** | **Upper Bound** |
| --- | --- | --- | --- | --- |
| **Model 1^†^** |  |  |  |  |
| Year of Survey | .052 (.006) | <.001 | .039 | .065 |
| Girl | .530 (.017) | <.001 | .497 | .562 |
| FAS | -.089 (.021) | <.001 | -.130 | -.048 |
| **Model 2 ^††^** |  |  |  |  |
| Year of Survey | .009 (.016) | .572 | -.022 | .039 |
| Girl | .436 (.045) | <.001 | .348 | .524 |
| FAS | -.206 (.055) | <.001 | -.314 | -.099 |
| Girl * Year | .029 (.013) | .026 | .003 | .054 |
| FAS * Year | .036 (.016) | .021 | .005 | .067 |
| B= unstandardized beta coefficient, S. E = standard error  **^†^**Model 1: main effects only. **^††^**Model 2: main effects, and interaction terms between sex and survey year, and between FAS and survey.  ***Model Fitness***: Model 1: R2 = .109, R2-change = .109; F(3, 9067) = 367.849, p<.001; Model 2: R2 = .110; R2-change = .001; F(5, 9065) = 222.952, p<.001 | | | | |
